# Effect of solid loading on the behaviour of pectin-degrading enzymes

**DOI:** 10.1186/s13068-021-01957-3

**Published:** 2021-04-28

**Authors:** Fan Li, Loïc Foucat, Estelle Bonnin

**Affiliations:** 1 INRAE, UR 1268, Biopolymers Interactions Assemblies BIA, F-44316 Nantes, France; 2 School of Life Sciences, Northeast Normal University, Changchun, 130024 People’s Republic of China; 3 INRAE, BIBS facility, F-44316 Nantes, France

**Keywords:** High-solids loading, Glycoside hydrolases, Polygalacturonase, Pectin lyase, Water mobility

## Abstract

**Background:**

Pectin plays a role in the recalcitrance of plant biomass by affecting the accessibility of other cell wall components to enzymatic degradation. Elimination of pectin consequently has a positive impact on the saccharification of pectin-rich biomass. This work thus focused on the behaviour of different pectin-degrading enzymes in the presence of low (5%) to high (35%) solid loading of lemon peel.

**Results:**

High solid loading of lemon peel affected pectin solubilisation differently depending on the pectinase used. Pectin lyase was less sensitive to a reduction of water content than was a mixture of endopolygalacturonase and pectin methylesterase, regardless of whether or not the latter's mode of action is processive or not. Marked changes in water mobility were observed along with enzymatic degradation depending on the enzyme used. However, the pectin lyase resulted in less pronounced shifts in water distribution than polygalacturonase–pectin methylesterase mixtures. At similar pectin concentration, pectin solutions hindered the diffusion of hydrolases more than the solid substrate. This can be attributed to the high viscosity of the highly concentrated pectin solutions while the solid substrate may provide continuous diffusion paths through pores.

**Conclusions:**

The increase in solid substrate loading reduced the efficiency of pectin-degrading enzymes catalysing hydrolysis more significantly than those catalysing *β*-elimination. LF-NMR experiments highlighted the impact of solid loading on water mobility. Compared to other enzymes and whatever the solid loading, pectin lyase led to longer relaxation times linked with the most destructuration of the solid substrate. This new information could benefit the biorefinery processing of pectin-rich plant material when enzymes are used in the treatment.

## Background

Massive non-renewable energy consumption and environmental deterioration has encouraged the development of alternative energy sources [1]. In this context, second-generation bioenergy, defined as derived from lignocellulose that is not part of the human food supply chain (e.g. agro-industrial residues like bagasse, stover, husks and municipal solid waste) has been attracting increasing interest [2, 3]. Agricultural plant by-products, which mainly comprise cell wall polysaccharides, are recognised as the largest renewable source of sugars for conversion into biochemicals and biofuels. Enzymatic hydrolysis has long been studied with the aim of depolymerising plant cell wall polysaccharides, but is considered to be industrially uncompetitive due to the high costs. One way to reduce its costs is to decrease the water inputs by conducting the process at high solid loading, i.e. ≥ 15% w/w solid matter content [4]. Increased solid loading reduces the volume of the system, increases the concentration of the product, and thus reduces operating costs. However, high solid loading may have negative impact on the enzyme action. Previous studies on cellulases have shown that several possible mechanisms contribute to the so-called solids effect: product inhibition, lack of enzyme adsorption, limitation of enzyme and product diffusion, inefficient mixing, and water availability [5–8]. However, the effect of mixing is controversial as the saccharification yield of wheat straw in a horizontal drum was not affected by the mixing speed [9], although mechanical agitation was shown to reduce the activity of cellulolytic enzymes used for flax fibre attrition at incubation times longer than 24 h [10]. Not only the agitation speed, but also the design of the reactor and the way it is loaded paly crucial roles [11]. In this context, complementary approaches to reduce the high-solids effect have been suggested, including optimising the hydrolysis reactor, improving biomass degradability by pre-treatment, and adding the accessory enzymes to better degrade the substrate or eliminate inhibiting products [12–14].

In addition to cellulose, pectin is present in the cell wall and has been shown to play an important role in plant biomass recalcitrance [15–17]. The nature and structure of pectin and its role in cell wall architecture have a negative impact on the processing of plant biomass. The presence of pectin increases the viscosity of the enzymatic reaction medium, especially at high loadings. Adding pectin-degrading enzymes during biomass processing consequently greatly increased the release of glucose from pectin-containing lignocellulosic biomass by improving the access of cellulases to cellulose [15] and by reducing the viscosity of the reaction medium [18–20]. Pectin is also a major mediator of cell wall porosity. The elimination of pectin increases wall porosity thereby increasing diffusion rates and enzyme accessibility during biomass processing. In the literature, the positive effect of removing pectin on the use of plant biomass has also been demonstrated through genetic regulation of pectin synthesis in various feedstocks. Overexpressing a pectin-degrading enzyme in transgenic aspen increased the solubility of pectin and resulted in higher yields of fermentable sugars [21]. In switchgrass the downregulation of a gene involved in homogalacturonan (HG) biosynthesis improved sugar yield [22]. Pectinases are thus considered to play a crucial role in the efficient hydrolysis of pectin-rich lignocellulosic biomasses. Polygalacturonases and lyases catalyse the degradation of pectin through depolymerisation, while pectin methylesterases catalyse the de-esterification reaction. All these enzymes, plus others involved in the degradation of pectin were recently reviewed [23]. According to the CAZy database, pectin methylesterases (PME, EC 3.1.1.11) belong to the CE8 family [24]. They catalyse the specific demethylesterification of methyl esters at the C6 of galacturonic acid (GalA), releasing non-methyl esterified HG and methanol. Endo-polygalacturonases (PG, EC 3.2.1.15) all belong to the CAZy family GH28 and generally catalyse the hydrolysis of the α-(1–4) glycosidic bond between two adjacent d-GalA units in non-methylated stretches of HG. Pectin lyases (PL, EC 4.2.2.10) catalyse the β-elimination reaction in the methylated area of HG and produce unsaturated oligogalacturonides. Thus, as a hydrolase, PG requires a water molecule to catalyse the bond breakdown, whereas PL does not. PG and PL also have different specificities as PG cleave the linkage between two de-esterified GalA residues while PL recognises methylesterified GalA. For this reason, the depolymerisation ability of PL can be compared to that of a PG + PME mixture. The mechanism by which pectinases cleave glycosidic bonds has been extensively studied. However, how the enzymes deconstruct polysaccharides in conditions in which they lack of available water, which limits their mobility and hence the degradation reaction is still not well understood.

The aim of the present work was thus to investigate the behaviour of pectin-degrading enzymes at high solids loading. The depolymerisation action of pectinases was followed by the solubilisation of GalA and neutral sugars, and by analysing the degradation products. In parallel, water mobility was studied using low field-NMR (LF-NMR) ${T}_{2}$ relaxometry, to consider the relationship between enzyme efficiency and water dynamics.

## Results and discussion

### Effect of solid loading on GalA solubilisation

Lemon peel was prepared as an alcohol-insoluble residue (AIR) to isolate the cell walls. Determination of the monosaccharide composition by gas chromatography (Table 1) shows that lemon peel AIR contained about 47% pectin. The degree of methylation (DM) determined by colorimetry was 77 ± 0.5.

**Table 1:** Monosaccharide composition of the AIR prepared from lemon peel

Sugars	Percentage weight
Rhamnose	0.94 ± 0.04
Fucose	0.44 ± 0.01
Arabinose	7.72 ± 0.31
Xylose	3.88 ± 0.33
Mannose	4.03 ± 0.01
Galactose	6.91 ± 0.26
Glucose	23.56 ± 1.31
GalA	31.86 ± 1.54
Total	79.34 ± 3.81

The standard deviation corresponds to the duplicate analysis

**Fig. 1:**
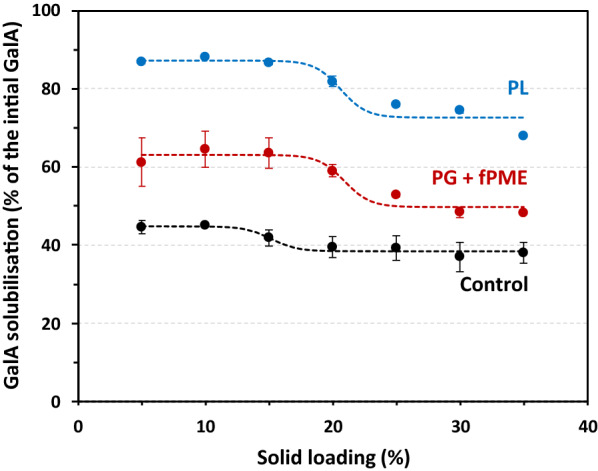
GalA solubilisation in lemon peel AIR after enzymatic treatment at different solid loadings. Treatments: pectin lyase (PL, blue), polygalacturonase + fungal pectin methylesterase (PG + fPME, red), buffer (control, black); solid loadings between 5 and 35%. Bar: standard error, *n* = 2. The dashed lines correspond to the sigmoidal fit of the experimental points

The AIR was hydrolysed by a mixture of PG + fPME (fungal PME) or by PL over a range of 5% to 35% solid loading. The amount of PG + fPME or PL was calculated according to the amount of GalA in the peel AIR to reach a similar enzyme-to-substrate ratio. The results were compared to the control incubation carried out in the absence of enzyme. As shown in Fig. 1, GalA was partially solubilised in the buffer alone, from 44 to 38% of the initial GalA content for solid loadings from 5 to 35%. Pectin from citrus peel is partly extractable with water [25] and the present extraction was favoured by the ionic strength of the buffer. The increase in dry matter content tended to have a negative impact on the solubilisation, probably linked to the physical constraints caused by congestion which limited the mass transfer. GalA solubilisation by the two enzyme treatments decreased with increasing solid loading, indicating that PG + fPME mixture and PL were both affected by the dry matter content of the reaction medium. The GalA solubilisation decreased from 63 to 48% with PG + fPME and from 87 to 67% with PL, whereas the decrease observed in the control was lower, from 44 to 38%. Since the same amount of enzyme units was used for PG and PL, PL degraded solid AIR more efficiently. As determined by a sigmoidal fit (s-shape), the inflection point was roughly the same in the two enzymatic treatments at about 21% solid loading, whereas in the absence of enzymes the inflection point occurred at a lower solid content (about 15%). Above 5% solid loading on both sides of these inflection points, solubilisation remained almost constant. In the literature, the impact of high solid loading is most often studied on cellulases and the mechanisms most frequently described explain the solids effect evolve linearly with the content of solid substrate [26]. However, an inflection point, as defined by the slope change, was observed in the same range of dry matter content on steam pre-treated wheat straw with different cellulase combinations [14]. Among other explanations, reduced hydrolysis efficiency has been attributed to a limited adsorption of the enzyme, hampered by the high concentration of substrate [9], or to an increase of the proportion of non-productive modes of adsorption [26].

**Fig. 2:**
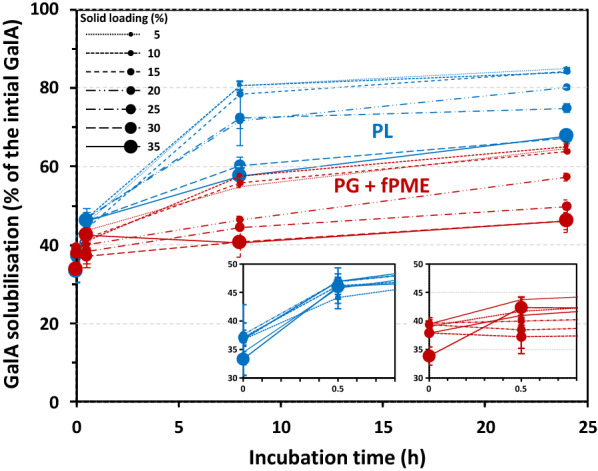
Time course of GalA solubilisation in the presence of enzymes at different solid loadings. Treatments: pectin lyase (PL, blue), polygalacturonase + fungal pectin methylesterase (PG + fPME, red); solid loadings between 5 and 35%. Bar: standard error, *n* = 2

We further monitored the time course of pectin degradation at the different solid loadings (Fig. 2). The curves of GalA solubilisation were characteristic of enzymatic degradation with a high initial rate during the first several hours and a subsequent slowdown. At all time points and in both treatments, the GalA solubilisation was similar at 5%, 10% and 15% solid loading samples. Like Fig. 1, this suggests that, up to 15%, dry matter content did not influence the efficiency of pectinolytic enzymes on solid substrates. Moreover, up to 15% solid loading, the mixture of PG + fPME and PL did not seem to be sensitive to the product inhibition. In fact, high concentrations of glucose have been shown to inhibit cellulase activity [27] and monomer GalA has been shown to inhibit *Aspergillus niger* polygalacturonase [28]. In the present study, the solid loadings tested made the conditions less favourable for enzyme action, and it is unlikely that a high amount of monomer was produced. Obviously, the limited mass transfer could result in a higher concentration of reaction products in the immediate vicinity of the enzymes, but it was impossible to access the local concentration of products.

### Effect of solid loading on the degradation products

**Fig. 3:**
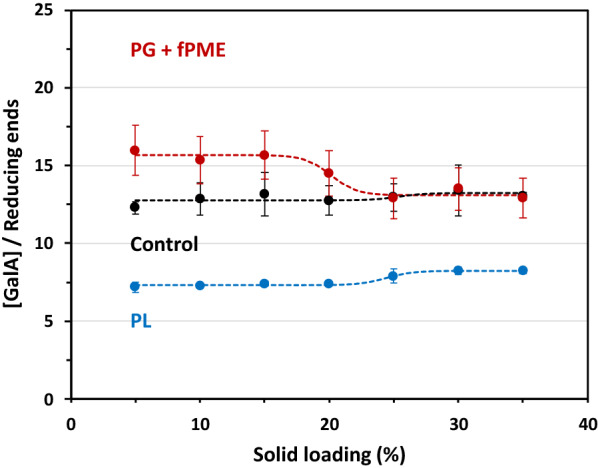
Average degrees of polymerisation of the degradation products. Treatments: pectin lyase (PL, blue), polygalacturonase + fungal pectin methylesterase (PG + fPME, red), buffer (Control, black)**;** solid loadings between 5 and 35%. Bar: standard error, *n* = 2. The dashed lines correspond to the sigmoidal fit of the experimental points

The average degree of polymerisation (DP) of the degradation products was calculated from the ratio [GalA]/[reducing ends]. It was about twice as high after PG + fPME treatment than after PL treatment (Fig. 3).

**Fig. 4:**
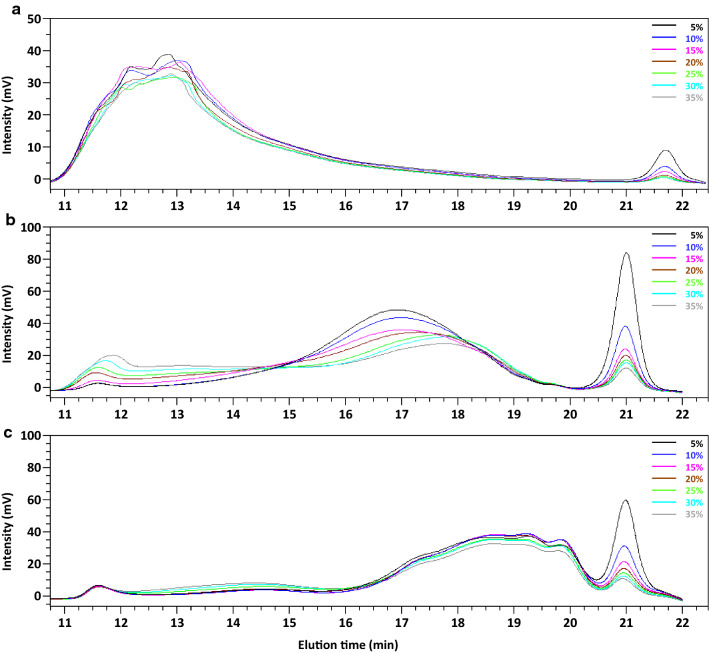
HPSEC profiles of the degradation products. Treatments: control samples (**a**), polygalacturonase + fungal pectin methylesterase (**b**), pectin lyase (**c**). In each graph, the different solid loadings from 5 to 35% are represented by different colours

The DP in the control did not change significantly with an increase in solid loading (DP = 12–13), like with PL, where the DP remained roughly constant (DP = 7–8), indicating that the substrate loadings had no effect on the DP of the degradation products. With PG + fPME, the average DP was higher than that in the control at 5% to 20% solid loading. This suggests that the products released by the enzyme combination had a much higher DP resulting in a higher average value than in the control. The DP of PG + PME products was also higher than that of PL products and decreased from 16 to 13 according to a sigmoidal curve when the solid content was increased from 5 to 35%. The respective values of average DP with PL and PG + fPME were consistent with the level of solubilisation: the higher the solubilisation (PL), the lower the average DP. With PG + fPME the curve showed an inflexion point at around 20% solid loading, in the same range as the curve for the GalA solubilisation (Fig. 1). In both cases, DP was higher than the expected limit DP. Indeed, the end products in the optimal conditions were mono- and disaccharides with PG, and di- and trisaccharides with PL [23, 29]. However, the calculated ratio is an average value of the final DP. For this reason, high-performance size exclusion chromatography (HPSEC) was used to investigate the molar mass distribution of the products (Fig. 4).

In the absence of enzyme (control, Fig. 4a), the material eluted in a large peak between 11 and 14 min. After PG + fPME treatment (Fig. 4b), three peaks eluted, one at the highest elution volume (21 min) corresponding to short oligosaccharides, one at the lowest elution volume (11–12 min) corresponding to the non-degraded fraction, and one at the intermediate elution volume (15–19 min). Increasing the substrate loading led to an increase in the non-degraded fraction, whereas the intermediate fraction and the small oligosaccharides decreased. This also confirmed the fact that the monosaccharide content was too low to inhibit the PG. The PL degradation also produced a 3-peak profile (Fig. 4c). The peak associated with the recalcitrant fraction (around 11 min) was the smallest. The intermediate fraction eluted at higher volumes than after PG + fPME treatment (17–20.5 min), demonstrating that DP was lower after PL treatment. The change in the chromatographic profiles of PL samples was much less marked with an increase in the solid loadings, showing that mass distribution was similar in all the PL-treated samples in agreement with the average DP (Fig. 3), whereas the mass distribution of PG + fPME-treated samples changed slightly resulting in a slight decrease in the average DP. The difference in the distribution of the products on the two series of samples suggests that PL was less sensitive to low water content than the PG + fPME mixture.

**Fig. 5:**
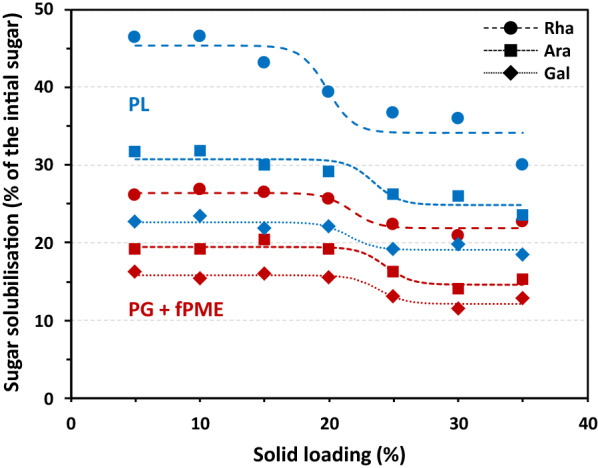
Neutral sugar solubilisation of lemon peel AIR after enzymatic degradation. Treatments: pectin lyase (PL, blue), polygalacturonase + fungal pectin methylesterase (PG + fPME, red); solid loadings between 5 and 35%. Results are expressed as a percentage of the initial concentration of each sugar in the starting material. Rhamnose, circle; arabinose, square; galactose, lozenge. The dashed lines correspond to the sigmoidal fit of the experimental points

Neutral sugars in the reaction products released by PG + fPME and PL were analysed by gas chromatography. The linkage breakdown in the homogalacturonic zones induced the solubilisation of the rhamnogalacturonan that carry the neutral sugars side chains. As expected the main neutral monosaccharides (rhamnose, arabinose and galactose) present in pectin were detected (Fig. 5). All the monomers followed an s-shape curve like the GalA solubilisation (Fig. 1): the solubilisation decreased over a narrow window and was more or less constant before and after. In both series of samples, the solubilisation of rhamnose was higher than that of arabinose, and of galactose, when expressed as a percentage of the initial content. PL released more neutral sugars in the soluble fraction than the PG + fPME mixture, which was consistent with the higher efficiency demonstrated with the GalA solubilisation.

Until now, most studies of high-solid loading systems have focussed on the use of cellulase to transform biomass. Since the target is the production of sugar, most of these studies used sugar conversion as the only indicator to investigate the effect of solid loading [4]. However, our results suggest that more complex phenomena are involved in the actual reaction systems under different solid loadings, which need to be investigated in more detail.

### Effect of PME mode of action on pectin degradation at high solid loading

PMEs are well known to have different modes of action, especially depending on whether they are of plant or fungal origin. Fungal PMEs use a non-processive mechanism and remove the methyl groups randomly, whereas the plant PMEs use a single-chain and multiple attack mechanism, thus behaving as a processive enzyme and releasing block-wise distributed free GalA [30–34].

**Fig. 6:**
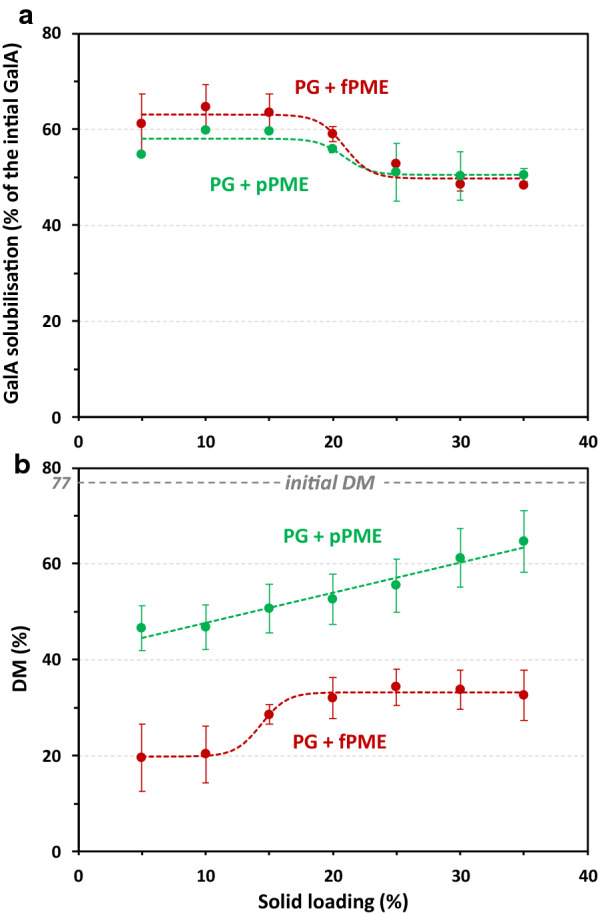
GalA solubilisation (**a**) and degree of methylation (**b**) of the hydrolysis products. Treatments: polygalacturonase + fungal pectin methylesterase (PG + fPME, red), polygalacturonase + plant pectin methylesterase (PG + pPME, green); solid loadings between 5 and 35%. Bar: standard error, *n* = 2. The dashed lines correspond to the sigmoidal or linear fit of the experimental points

Next, the enzyme mixtures PG + fPME and PG + pPME prepared with the same enzymatic activity were compared in the hydrolysis of the lemon peel AIR between 5 and 35% solid loading. The solubilisation of GalA and the degree of methylation were analysed in the solubilised fraction (Fig. 6). The solubilisation of GalA in the presence of the two enzyme mixtures started in the same range (about 60% of the initial GalA) and followed the same pattern as that shown previously (Fig. 6a). At from 5 to 15% solid loading, the PG + pPME mixture tended to solubilise less GalA than the PG + fPME mixture, but the differences were not significant. Conversely, along the whole range of solid loading tested, the DM after PG + pPME treatment was about twice as high as than DM after PG + fPME (Fig. 6b), suggesting that fPME was more efficient in these conditions and explaining the lower GalA solubilisation with the PG + pPME mixture. Moreover, in the presence of pPME, the DM of the products increased linearly with an increase in solid loading. Even if in both cases the DM was lower than its initial value (DM = 77), these results suggest that the hindrance caused by the high concentration of solids in the diffusion and adsorption steps had a greater impact on pPME.

**Fig. 7:**
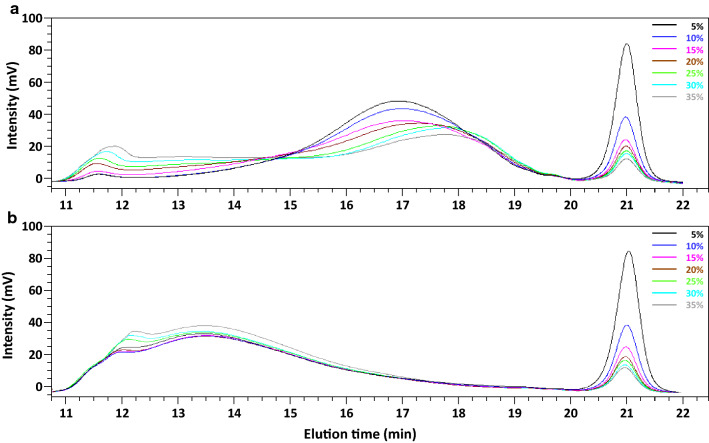
HPSEC profiles of the enzymatic hydrolysis products. Treatments: polygalacturonase + fungal pectin methylesterase (**a**), polygalacturonase + plant pectin methylesterase (**b**). In each graph, the different solid loadings from 5 to 35% are represented by different colours

The HPSEC product profiles showed that the main fraction after PG + pPME treatment had a larger DP, and that the distribution was roughly the same at for the different loadings (Fig. 7). This was quite different from the dispersed distribution observed after PG + fPME treatment at high solid loadings. Neutral sugars in the hydrolysis products were rather similar in the two series of experiments (data not shown). Thus, when PME was associated with PG to degrade a solid substrate, pPME allowed the release of a larger amount of longer pectin fragments than fPME.

### Water constraint profiles

**Fig. 8:**
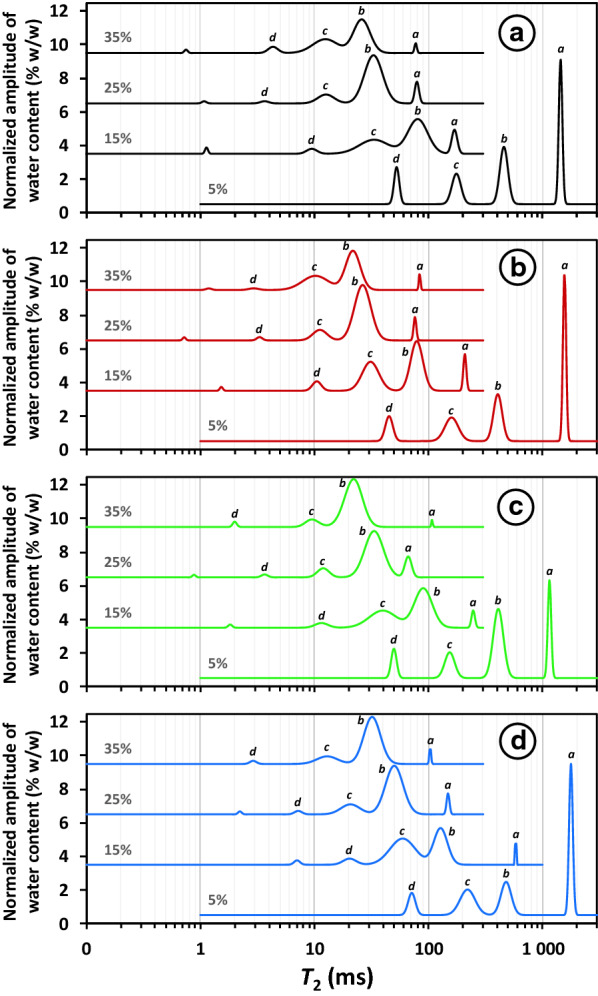
${T}_{2}$ water profiles of the control lemon peel AIR and after enzymatic degradation. Treatments: control samples (**a**), polygalacturonase + fungal pectin methylesterase (**b**), polygalacturonase + plant pectin methylesterase (**c**), and pectin lyase (**d**); solid loadings between 5 and 35%. Profiles are representative of triplicate samples

Water plays an important role in enzymatic degradation, notably as a diffusion medium for enzyme and products, and as a reactant in the catalysis for hydrolases, but not for lyases. In this work, water mobility distribution in whole lemon peel samples was characterised by LF-NMR to investigate changes induced by pectinolytic enzyme and solid loadings. Water constraint indicates the degree to which water is tightly bound to biomass and the LF-NMR experiments make it possible to distinguish several water populations according to their environment and associated mobility. Whatever the solid loading between 5 and 35%, four main distinct ${T}_{2}$ peaks were displayed (Fig. 8) and denoted by letters *a* for the longest relaxation to *d* for the shortest. The proportion of water (${P}_{2}$) was determined for each ${T}_{2}$, and is expressed in absolute water content (% weight/weight). The populations associated with more or less constrained water pools represent four physically and chemically distinct environments in the samples. In most of the samples, a fifth component was discriminated at very short ${T}_{2}$ of around 1 ms, but as this represented a very low population (< 1%), the associated relaxation time was poorly defined. Figure 8 is representative of each ${T}_{2}$ distribution obtained in triplicate. The changes in the water profiles as a function of solid loadings showed that the water constraints increased with solid loading. The associated shift towards shorter ${T}_{2}$ values was much greater between 5 and 15%, and 15% and 25% than between 25 and 35%, suggesting marked changes in water dynamics around 20% solid loading. This behaviour was supported by the rapid drop in GalA solubilisation shown in Fig. 1 (inflection points around 20%).

At 5% solid loading in the absence of enzyme (Control sample), the longest relaxation time ${T}_{2a}$ (≈ 1500 ms) accounted for about 40% (${P}_{2a}$) of the total water content (Table 2).

**Table 2:** ${T}_{2}$ relaxation times and ${P}_{2}$ populations measured for control lemon peel AIR and after enzymatic degradation. Treatments: control samples, polygalacturonase + fungal pectin methylesterase (PG + fPME), polygalacturonase + plant pectin methylesterase (PG + pPME), and pectin lyase (PL)

		*T*_2*i*_ (ms)								*P*_2*i*_ (% w/w)							
Solid loading	Sample	*T*_2*a*_		*T*_2*b*_		*T*_2*c*_		*T*_2*d*_		*P*_2*a*_		*P*_2*b*_		*P*_2*c*_		*P*_2*d*_	
5%	Control	1524	(238)	439	(24)	178	(2)	54	(1)	37	(3)	27	(4)	19	(1)	12.6	(0.4)
	PG + fPME	1478	(86)	428	(25)	159	(18)	44	(5)	34	(2)	28	(2)	20	(1)	13	(1)
	PG + pPME	1096	(159)	383	(35)	133	(29)	51	(8)	27	(5)	44	(2)	14	(2)	10	(3)
	PL	1776	(21)	439	(41)	199	(20)	66	(5)	39	(3)	23	(1)	23	(2)	10	(1)
15%	Control	180	(24)	83	(9)	35	(6)	10	(2)	7	(3)	44	(6)	29	(8)	4	(1)
	PG + fPME	216	(11)	77	(1)	30	(1)	10.2	(0.3)	8	(2)	40	(2)	31	(1)	5	(1)
	PG + pPME	236	(16)	90.1	(0.1)	40	(1)	12	(1)	5	(2)	44	(2)	30	(3)	3.9	(0.3)
	PL	497	(75)	125	(6)	59	(4)	20	(1)	3.0	(0.4)	33	(5)	43	(4)	5	(2)
25%	Control	75	(9)	33	(2)	12	(1)	3.4	(0.4)	7	(1)	57	(1)	9	(2)	1.2	(0.1)
	PG + fPME	78	(2)	27	(1)	12	(1)	3	(1)	5	(1)	56	(6)	13	(5)	1.0	(0.2)
	PG + pPME	65	(10)	33	(3)	11	(1)	3.5	(0.2)	13	(6)	54	(4)	6	(1)	1.1	(0.1)
	PL	145	(6)	50	(1)	20	(1)	8	(1)	4.1	(0.3)	57	(1)	12	(1)	2	(1)
35%	Control	73	(4)	25	(3)	11	(1)	4	(1)	1.8	(0.3)	40	(4)	20	(3)	3	(1)
	PG + fPME	83	(5)	21	(1)	9.9	(0.3)	2.8	(0.2)	1.9	(0.2)	39	(2)	22	(2)	1.1	(0.1)
	PG + pPME	100	(18)	22.0	(0.1)	9.4	(0.3)	2.3	(0.3)	0.8	(0.2)	57	(1)	6	(1)	1.4	(0.1)
	PL	107	(6)	32	(1)	13	(1)	2.7	(0.4)	1.9	(0.2)	52	(1)	10	(1)	1.3	(0.2)

Values in parentheses are standard errors, *n* = 3

Since ${T}_{2}$ relaxation time of pure water is about 4 s at the working temperature (40 °C), this “bulk” water was considered as “free-like”, but of restricted mobility due to the intrinsic viscosity of the sample. The longest ${T}_{2a}$ component was also observed in the three enzyme-treated samples, with a significantly lower value in PG + pPME treated samples, corroborated by a significantly lower longitudinal relaxation time ${T}_{1}$ value (data not shown). Whatever the sample considered, an increase in water constraint in all four pools was observed with an increase in solid loading, identified through decreasing relaxation times. This could be attributed either to increases in the viscosity of the medium, or to changes in the chemical environments experienced by water inside the sample, such as increased water-(macro)molecules interactions.

Compared with 15% dry matter loading, the change of the structure resulted in a shift in ${T}_{2}$ towards lower values (Table 2). The component associated with ‘bulk’ water ${T}_{2a}$ and ${P}_{2a}$ decreased dramatically. The two intermediate components ${T}_{2b}$ and ${T}_{2c}$ decreased concomitantly, whereas ${P}_{2b}$ and ${P}_{2c}$ increased to become the main water populations. Interestingly the ${T}_{2}$ values of the four pools in the PL-treated samples were significantly higher than those in the other samples. When solid loading increased at 25% and 35%, the most constrained pool remained almost unchanged among the samples, both in mobility (${T}_{2d}$) and in proportion (${P}_{2d}$). At 25% ${P}_{2b}$ became the main population at more than 50%. The corresponding ${T}_{2b}$ was around 80–90 ms at 15% and dropped down to about 30 ms at 25% and about 20 ms at 35%. The sum of the two intermediate pools ${P}_{2b}$ and ${P}_{2c}$ was almost constant at 64% ± 4% w/w, although the water content decreased by 10% between the 25%- and the 35%-series. Again, the PL-treated samples were an exception as ${T}_{2b}$ decreased from 125 ms at 15% to 32 ms at 35%. This result was attributed to the more efficient degradation of pectin by PL, which led to higher GalA solubilisation (Fig. 1), and thus limited the constraint of the solid substrate on water diffusion.

${T}_{2}$ time and water populations in PG + fPME samples were close to those in the control samples, while PG + pPME samples differed significantly from the other groups in ${T}_{2}$ time and in the proportions of water. *Aspergillus aculeatus* PG was previously shown to release oligosaccharides of different lengths depending on the distribution of residual methyl groups after PME treatment [34]. Methylation distribution was suspected to result in different water distribution in the system. Random demethylesterification by fPME made the distribution of residual methyl groups statistically homogenous, like that in the control samples. Conversely with pPME, processive mechanism led to a methyl distribution with long zones of free GalA, which could be the location of ‘clusters’ of water molecules. The means of observation used here distinguished water populations with different mobilities, but without being able to differentiate the nature of the chemical environments with which these different populations are associated. It is thus not possible to reasonably attribute the populations ${P}_{2}$ to specific molecular interactions or locations [35].

In previous studies, NMR was used to investigate biomass–water interactions in pure cellulose or lignocellulose by measuring the relaxation time of water protons. Felby et al. [36] used time domain NMR and showed that the state and location of water associated with cellulose in filter paper can be linked to the structural changes in the fibres during enzymatic degradation by cellulases. Selig et al. [8] studied the hydration and hydrolysis of cellulose Iβ, II, and III_I_ at increasing solids loadings up to 30% and suggested that cellulose III_I_ had the most restricted pool and that the changes in water distribution during enzymatic saccharification were more dramatic in cellulose III_I_ than in celluloses Ib and II. More recently, Weiss et al. [14] and Thomsen et al. [37] investigated the effect of substrate modifications under enzymatic degradation by commercial cellulases on the biomass–water interactions on wheat straw and spruce. Their results showed that the notable effect of solids was due to both enzyme and substrate, and that increased water constraint by the biomass correlated with higher hydrolysis yields. In the present work, the LF-NMR was measured on the whole reaction medium, which was composed of both the solid and the solubilised parts of the plant material. This may explain the different observations between water constraint and hydrolysis yield. When the solid content was the lowest (5%), the degradation was the highest and hence the soluble matter content was also the highest. The highest values of ${T}_{2}$ in the lowest solid loading samples were also assumed to be induced by the presence of higher proportions of the soluble fraction (see Figs. 1, 6a). The NMR analyses were performed on the whole reaction medium without separating the liquid and solid fractions to investigate the water constraints as they were in the reaction medium where the enzyme worked. However, it would be interesting to perform more NMR analyses to dissociate the respective contributions of the solid and the soluble parts.

### Effect of high substrate concentration in pectin solution

In the lemon peel AIR used as substrate in the above experiments, the pectin was entrapped in a solid matrix made of other cell wall polymers (Table 1). In the presence of the enzymes, this resulted in catalysis in the heterogeneous phase with a soluble enzyme and a solid substrate. In this section, isolated lemon pectin was used as a soluble and continuous viscous medium to investigate the impact of the substrate physical state of the substrate on the behaviour of the enzyme. Pectin solutions were prepared at 1.9%, 5.6%, 9.4% and 13.2% and corresponded to the pectin concentrations in AIR at 5%, 15%, 25% and 35%, respectively. The conditions used for the enzymatic treatments and detection methods were the same as previously.

**Fig. 9:**
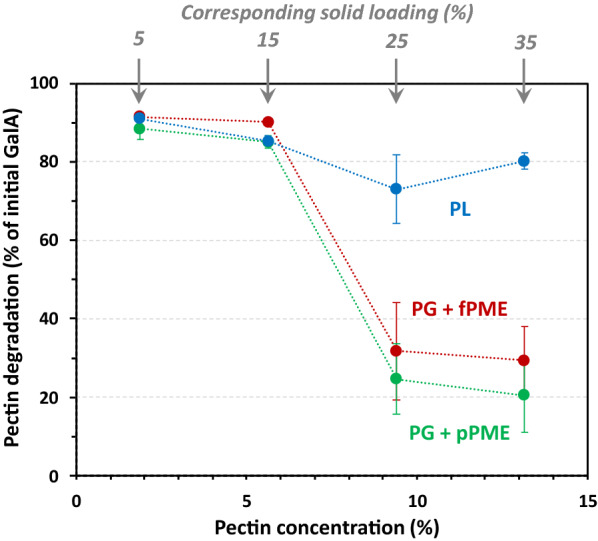
Degradation of pectin solution after enzymatic treatment at different substrate concentrations. Treatments: polygalacturonase + fungal pectin methylesterase (PG + fPME, red), polygalacturonase + plant pectin methylesterase (PG + pPME, green), pectin lyase (PL, blue); substrate concentrations between 1.9% and 13.2% pectin corresponding to 5% to 35% AIR loadings. Bar: standard error, *n* = 2

In the presence of PL, the GalA solubilisation decreased slightly from 90 to 80% with an increase in the substrate concentration (Fig. 9), which was consistent with the results obtained when AIR was used as the substrate (Fig. 1). Conversely, when PG was associated with fPME or pPME, the GalA release dropped dramatically between 5.6% and 9.4% pectin, and was finally much lower than with AIR at the highest substrate concentrations (25% and 35%, Fig. 6a). The molar mass distribution of the degradation products was consistent with those obtained with AIR (data not shown). All the results indicated that physical state of the pectin substrate affects its degradation. Indeed, the continuous medium produced by a high concentration of pectin was very viscous, which limited the diffusion of water and consequently the diffusion of enzymes. Although increasing the solid content reduces the enzyme diffusivity [7], the viscous soluble reaction medium further limited the diffusion of the enzymes. It hindered the enzyme diffusion more than solid AIR, where the solid matrix forms pores that may be interconnected and thus form preferential paths for the diffusion of solutes. However, it should be noted that the same limitation was not observed with PL, suggesting that the impeded diffusion of water was not the only phenomenon that occurs in these conditions. In the case of PG + fPME or PG + pPME, the observed pectin hydrolysis was the result of the action of two different enzymes that both require a molecule of water in their catalysis reaction. For family 1 glycoside hydrolases (GH1) [24], two water channels have been demonstrated from the protein surface to the protein core and a third channel goes down to the bottom of the active site cleft [38]. These channels were assumed to be fed by the surface of the protein, and the water forming the third channel was assumed to be involved in the catalysis. As a consequence, the availability of water molecules is of prime importance for these enzymes. Even if PGs belong to family GH28 and not to family GH1, it is tempting to speculate that the existence of water channels is a common feature of glycoside hydrolases. Conversely, PL does not require any water molecule for the catalysis and was thus less sensitive to the lack of water in the vicinity of the enzyme molecule.

## Conclusion

The high solids loading of lemon peel resulted in different decreases in GalA solubilisation depending on the pectinase used. PL was less sensitive to the low water availability, linked to its β-elimination mechanism. LF-NMR experiments highlighted the impact of solid loading on water mobility. After PL treatment, relaxation times were the longest regardless of the dry matter content. As this enzyme is the most efficient degrader of lemon peel, it led to greater destructuring of the solid, and hence in a greater water mobility. At similar pectin concentrations, the viscous medium limited enzyme diffusion even more than the solid substrate. Despite the variability of the structure of pectin depending on to the source, the results obtained here can be extrapolated to other plant materials because the pectic patterns recognised by the enzymes are always more or less present. Taken together, these findings could enable the development of new enzymatic biorefinery processes by considering the need for pectin degradation, particularly using PL.

## Materials and methods

### Preparation of alcohol-insoluble residue (AIR)

Dry lemon peel was kindly provided by CP Kelco (Denmark) and ground twice in a grid crusher (Gondard & Cie) to reach 1 mm particle size. The dry peel was then immersed in boiling 70% ethanol for 30 min. After filtration, the material was washed at the same ethanol concentration at room temperature until sugar was no longer detected in the filtrate. After another two washes in 96% ethanol, the sample was washed in acetone in glass G3 filters and then dried at 40 °C overnight.

### Enzymes

Endo-polygalacturonase (PG) was purchased from *Aspergillus aculeatus* (PG, Megazyme, Product code E-PGALUSP, Lot 00,304, see https://www.megazyme.com for more information). The pectin lyase (PL) was purchased from *Aspergillus niger* and purified in the laboratory as described elsewhere [28]. Two pectin methylesterases were used, one obtained from *Aspergillus aculeatus* (fPME, Novozymes, PPJ 4300) [39] and the other one was from orange peel (pPME, Sigma P0764). Solid enzymes were solubilised in acetate buffer (100 mM, pH 5.5). All the enzymes were diluted as required with the same buffer.

### Enzymatic incubation of AIR and recovery of the hydrolysis products

Four mL acetate buffer (100 mM, pH 5.5) or enzyme solution was poured into a centrifuge tube, and the required amount of AIR was added to make a series of incubations with 5%, 10%, 15%, 20%, 25%, 30% and 35% solid loading. The final concentrations of PG, PL and PME were 126 nkat/g AIR, 126 nkat/g AIR and 91 nkat/g AIR, respectively. The reaction mixture was incubated at 40 °C on a shaking plate at 200 rpm for 17 h. To extract the soluble part of the samples, 12 mL water was added, and the samples were centrifuged at 20,000 rpm for 10 min. The supernatant was boiled for 10 min to inhibit enzyme activity, and then stored at − 20 °C until further analysis. Each experiment was carried out in duplicate.

### GalA and reducing ends

The GalA content in the supernatant was measured using the automated colorimetric m-hydroxybiphenyl method [40]. Reducing end assay was performed using the Nelson method adapted to microplates [41]. Each determination was carried out in duplicate.

### HPSEC

High-performance size exclusion chromatography (HPSEC) was performed using column ShodexOH-Pack SB-804 HQ column eluted at 0.5 mL/min with 50 mM sodium nitrate at room temperature. The column effluent was monitored using a differential refractometer (Erma 7512, Japan). The enzymatic hydrolysates were diluted to reach a sugar concentration of 2.5 mg/mL as the sample.

### Neutral sugar composition

Individual neutral sugars were determined by gas–liquid chromatography using a BP-225 fused-silica capillary column (SGE, Australia; 25 m × 0.32 mm) mounted on a Perkin-Elmer autosystem gas chromatograph. The degradation supernatant was first hydrolysed in 2 M H_2_SO_4_ at 120 °C for 3 h, and then derivatized in alditol acetates to be the sample [42]. Each determination was carried out in duplicate.

### Degree of methylation (DM)

The methyl esters were saponified in a sodium hydroxide medium, and then quantified with the colorimetric method using N-methylbenzothiazolinone-2-hydrazone (Sigma M8006) and alcohol oxidase from *P. pastoris* (E.C.1.1.3.13, Sigma A2404) for oxidation of the released methanol [43]. GalA determination was used to calculate the DM, which is the molar ratio of methanol to GalA. Each determination was carried out in duplicate.

### Low field-nuclear magnetic resonance (LF-NMR)

The required amount of lemon peel AIR was mixed with buffer or enzyme solution in an 18-mm-diameter NMR tube to reach solid loading of 5%, 15%, 25% and 35% in a sample of approximately 1 cm in height. All the samples were prepared in triplicate and incubated at 40 °C for 24 h. LF-NMR analyses were carried out on the whole reaction medium using a Bruker Minispec mq20 spectrometer operating at 0.47 T (20 MHz proton resonance frequency) equipped with a thermostated (± 0.1 °C) ^1^H probe. Before NMR data acquisition, the sample was left 10 min in the spectrometer to allow the temperature to stabilise at 40 °C. The transverse ${T}_{2}$ relaxation curves were acquired using a Carr–Purcell–Meiboom–Gill (CPMG) sequence. Depending on the percentage of solid loading, the 180° pulse separation was 0.25 (35% DM) to 2 ms (5% DM), 1000 to 2500 even echoes were collected and 128 to 64 scans were acquired with a recycle delay of 3 to 15 s, resulting in a total acquisition time of between, 6 and 15 min. An inverse laplace transformation (ILT) was applied to convert the relaxation signal into a continuous distribution of ${T}_{2}$ relaxation components. A numerical optimisation method was used by including non-negativity constraints and L1 regularisation and by applying a convex optimisation solver primal–dual interior method for convex objectives (PDCO) [44, 45]. To extract quantitative information from the continuous analysis, ${T}_{2}$ distribution envelopes were modelled using a sum of peaks with a normal asymmetrical shape using an in-house routine developed in Matlab® software. Relative peak area, peak width/dispersion and ${T}_{2}$ values of each peak were determined in this way.

### Enzymatic degradation on pectin solution

Native citrus pectin was kindly provided by Cargill (Beaupte, France). Pectin solutions were prepared at high concentrations to reach the same GalA concentrations as in the AIR samples. Citrus pectin was dissolved in acetate buffer (100 mM, pH 5.5) at a concentration of 1.9%. The amount of GalA in this pectin solution was equivalent to that in 5% AIR substrate. The solution was further dialysed several times against polyethylene glycol 35,000 (PEG, Sigma) solutions prepared at 5.6%, 9.4% and 13.2%, respectively, to reach 5.6%, 9.4% and 13.2% GalA concentrations. The GalA content was then equivalent to that in AIR at 15%, 25% and 35%, respectively. The conditions for the enzymatic treatment of the solutions were similar to those for AIR. After incubation, 3 mL water was added and the slurry was vigorously vortexed to extract the degraded material. After centrifugation, the supernatant was boiled for 5 min to stop enzyme activity. Each experiment was carried out in duplicate.

## Data Availability

Almost all data generated or analysed during this study are included in this published article. The ‘data not shown’ are available from the corresponding author on reasonable request.

## Abbreviations

AIR: Alcohol-insoluble residue
DP: Degree of polymerisation
GalA: Galacturonic acid
GH: Glycoside hydrolases
HPSEC: High-performance size exclusion chromatography
LF-NMR: Low field-nuclear magnetic resonance
PG: Endo-polygalacturonase
PL: Pectin lyase
PME: Pectin methylesterase (fungal PME, fPME; plant PME, pPME)
